# COVID-19 lockdowns show reduced pollution on snow and ice in the Indus River Basin

**DOI:** 10.1073/pnas.2101174118

**Published:** 2021-04-26

**Authors:** Edward Bair, Timbo Stillinger, Karl Rittger, McKenzie Skiles

**Affiliations:** ^a^Earth Research Institute, University of California, Santa Barbara, CA 93106-3060;; ^b^Institute for Arctic and Alpine Research, University of Colorado, Boulder, CO 80309-0450;; ^c^Department of Geography, University of Utah, Salt Lake City, UT 84112

**Keywords:** remote sensing, Indus, COVID-19, snow, light-absorbing particles

## Abstract

Melting snow and ice supply water for nearly 2 billion people [J. S. Mankin, D. Viviroli, D. Singh, A. Y. Hoekstra, N. S. Diffenbaugh, *Environ. Res. Lett.* 10, 114016 (2015)]. The Indus River in South Asia alone supplies water for over 300 million people [S. I. Khan, T. E. Adams, “Introduction of Indus River Basin: Water security and sustainability” in *Indus River Basin*, pp. 3−16 (2019)]. When light-absorbing particles (LAP) darken the snow/ice surfaces, melt is accelerated, affecting the timing of runoff. In the Indus, dust and black carbon degrade the snow/ice albedos [S. M. Skiles, M. Flanner, J. M. Cook, M. Dumont, T. H. Painter, *Nat. Clim. Chang.* 8, 964−971 (2018)]. During the COVID-19 lockdowns of 2020, air quality visibly improved across cities worldwide, for example, Delhi, India, potentially reducing deposition of dark aerosols on snow and ice. Mean values from two remotely sensed approaches show 2020 as having one of the cleanest snow/ice surfaces on record in the past two decades. A 30% LAP reduction in the spring and summer of 2020 affected the timing of 6.6 km^3^ of melt water. It remains to be seen whether there will be significant reductions in pollution post−COVID-19, but these results offer a glimpse of the link between pollution and the timing of water supply for billions of people. By causing more solar radiation to be reflected, cleaner snow/ice could mitigate climate change effects by delaying melt onset and extending snow cover duration.

Snow and ice melt supply water to nearly 2 billion people (1). The Indus River in South Asia alone (Fig. 1) supplies water to over 300 million people (2). Multiple studies of the region find dust and soot on the snow/ice surface in sufficient quantities to degrade albedo (3, 4).* These light-absorbing particles (LAP) reduce albedo in the visible to near-infrared wavelengths, causing more solar radiation to be absorbed, which, in turn, causes grain growth and additional absorption in the near-infrared and shortwave infrared. Polluted snow causes earlier and faster melt, upsetting the snow albedo feedback, an important component of Earth's climate. Conversely, cleaner snow counteracts this climate change effect by delaying the onset of melt and extending snow cover duration.

**Fig. 1.**
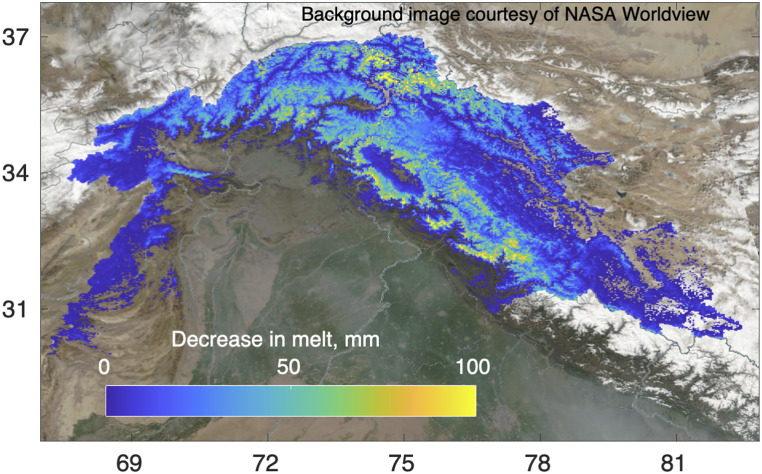
Decrease in melt from an energy balance model using observed snow/ice cover in 2020 compared to the same snow/ice cover with 20-y mean levels of LAP. Shown is the mean difference using approaches 1 and 2 (see *Materials and Methods*).

The COVID-19 epidemic has resulted in decreased emissions, especially in the spring of 2020 when many countries were under strict stay at home orders. Air quality in cities worldwide, for example, Delhi, India (5), improved dramatically. It's been unclear how the epidemic has affected pollution on the snow, and therefore the water supply for billions of people. Here, we use two remote sensing approaches to examine pollution anomalies, compared to the past 20 y, on the snow surface during the spring and summer of 2020 in the Indus River Basin.

## Results

**Fig. 2.**
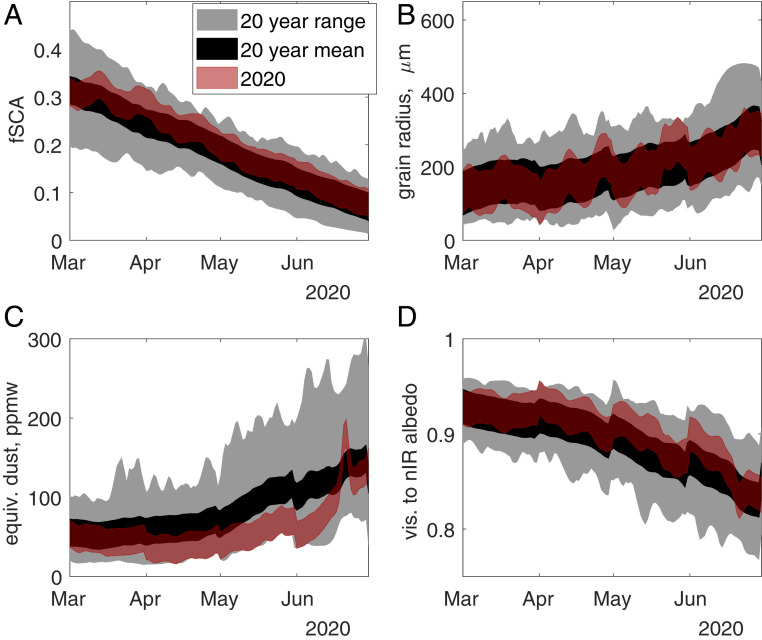
Results from approach 1: (*A*) fractional snow-covered area (fsca) over the Indus; (*B*) snow grain radius, in micrometers; (*C*) equivalent dust concentration (equiv. dust), in ppmw; and (*D*) visible (vis.) to near-infrared (nIR) snow albedo. For the red and black lines, line width represents uncertainty. The timescale shown is the melt season, with times after July not shown, due to cloud obfuscation of the snowpack from the monsoon.

**Fig. 3.**
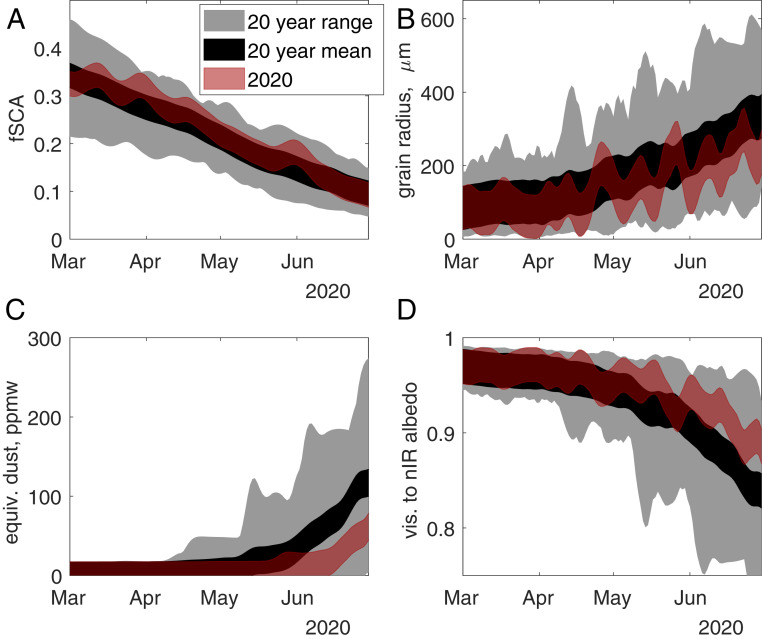
Results from approach 2. *A*−*D* are the same as in Fig. 2.

Using two remote sensing approaches (6–8), snow-covered area, grain size, equivalent dust concentration (a proxy for all LAP), and visible to near-infrared snow albedo were estimated (Figs. 2 and 3). The snow was significantly cleaner in 2020 than mean values over the past 20 y, and showed some of the cleanest measurements on record (Figs. 2*C* and 3*C*). Relative to the annual mean, approach 1 shows a decrease of 22 parts per million by weight (ppmw) of equivalent dust (39% of the annual mean), while approach 2 shows a decrease of 21 ppmw (22% of the annual mean).

There were differences between the approaches for the magnitude of LAP concentration, with approach 1 showing overall higher concentrations and a minimum around 33 ppmw, while approach 2 showed lower concentrations and negligible minimum concentration. The snow-covered area and the snow grain size were not significantly different in 2020 than the mean (red lines and black lines always intersect) for both approaches (Figs. 2 *A* and *B* and 3 *A* and *B*). Visible to near-infrared albedos (Figs. 2*D* and 3*D*) were above the mean values for both approaches, and were significantly brighter for almost the whole month of June for approach 2. Grain size and LAP affect albedo in this visible to near-infrared range, especially for Moderate Resolution Imaging Spectroradiometer (MODIS) band 2 (0.841 µm to 0.876 µm). These overlapping effects and a grain size similar to previous years may explain why visible to near-infrared albedos are not significantly brighter in approach 1 or for times prior to June in approach 2.

An energy balance model, forced with snow cover from approaches 1 and 2, was used to model melt using the observed cleaner snow compared to simulated dirtier snow with mean 20-y LAP concentration. Compared to the dirtier snow, the average volume of melt retained in 2020 is 6.55 km^3^.^†^

## Conclusion

Two independent approaches show significant decreases in LAP over the Indus Basin in 2020. This decrease was presumably caused by the COVID-19 lockdowns and associated decreased economic activity, but a more thorough analysis, for example, with in situ measurements of pollutant composition, would be needed to establish causality. Assuming the lockdowns were the cause, this study demonstrates how changes in human behavior can affect the water supply for billions of people.

## Materials and Methods

Approach 1 is the MODIS Snow-Covered Area and Grain Size (8) plus Dust and Radiative Forcing in Snow (7), interpolated and smoothed (9) to account for clouds and off-nadir views. Approach 2 is the Snow Property Inversion from Remote Sensing (6), which accounts for snow containing LAPs and also provides interpolated and smoothed results. Both approaches show high accuracy and nearly unbiased results for fractional snow-covered area (6, 10), with summed basin-wide RMSE values of 5.8% and 5.1% for approaches 1 and 2, respectively. Grain size and LAP concentration have not yet been validated in approach 2, but were validated with over 1,800 d of radiometer measurements at three sites in approach 1 (11), with grain sizes showing an RMSE of 118 µm and 3.6% RMSE in visible to near-infrared (0.350 µm to 0.876 µm) albedo reduction (called “deltavis”). The deltavis can be converted to an effective dust or black carbon concentration, comparable to the LAP concentration from approach 2, using a radiative transfer model (11), which makes the 3.6% RMSE in deltavis equivalent to 34 ppmw dust or 850 parts per billion by weight of black carbon. Because black carbon, dust, and other LAPs are usually spectrally inseparable with multispectral sensors (6), the effective dust concentration encompasses effects from all dark pollutants. Because it's the most common aerosol (4), an effective dust concentration is used, even though black carbon levels may have changed more than dust during the COVID-19 lockdowns. The RMSE values above were used to represent uncertainty (red line width) in Figs. 2 and 3.

The ParBal model is detailed in previous publications (12, 13). To model the effect of the cleaner snowpack in 2020, ParBal was run over the Indus Basin (14), with the snow surface estimated from approaches 1 and 2. Then, the observed decrease in LAP (compared to the 20-y mean) was added to estimate the change in melt magnitude from the cleaner snow.

The snow cover time space data cubes are available at ftp://snowserver.colorado.edu/pub/fromRittger/products/Indus and ftp://ftp.snow.ucsb.edu/pub/org/snow/products/SPIRES/Indus/. The source codes for approach 1 are not publicly available. The source codes for approach 2 and the ParBal model are at https://github.com/edwardbair/SPIRES/releases/tag/v1.0 and https://github.com/edwardbair/ParBal/releases/tag/v1.0.

## Data Availability

The time space cubes with the snow cover variables covering the Indus are available at ftp://snowserver.colorado.edu/pub/fromRittger/products/Indus (15) and ftp://ftp.snow.ucsb.edu/pub/org/snow/products/SPIRES/Indus/ (16). The source codes for approach 1 are not publicly available. The source codes for approach 2 and the ParBal model are available in open source repositories at https://github.com/edwardbair/SPIRES/releases/tag/v1.0 (17) and https://github.com/edwardbair/ParBal/releases/tag/v1.0) (18).

## Footnotes

*Hereafter, for brevity, we only refer to the snow surface rather than the snow/ice surface, as this part of the world contains much more snow on top of ice or dry ground/vegetation than exposed glacier ice (5).

^†^This snow would have stayed frozen in the basin.
